# Unconventional Biocatalytic Approaches to the Synthesis of Chiral Sulfoxides

**DOI:** 10.1002/cbic.202000430

**Published:** 2020-09-11

**Authors:** Silvia Anselmi, Nandini Aggarwal, Thomas S. Moody, Daniele Castagnolo

**Affiliations:** ^1^ School of Cancer and Pharmaceutical Sciences King's College London 150 Stamford Street London SE1 9NH UK; ^2^ Almac Sciences 20 Seagoe Industrial Estate Craigavon BT63 5QD UK; ^3^ Arran Chemical Company Limited Unit 1 Monksland Industrial Estate, Athlone, Co. Roscommon N37 DN24 Ireland

**Keywords:** biocatalysis, deep eutectics, ionic liquids, reductive enzymes, sulfoxides

## Abstract

Sulfoxides are a class of organic compounds that find wide application in medicinal and organic chemistry. Several biocatalytic approaches have been developed to synthesise enantioenriched sulfoxides, mainly by exploiting oxidative enzymes. Recently, the use of reductive enzymes such as Msr and Dms has emerged as a new, alternative method to obtain enantiopure sulfoxides from racemic mixtures. In parallel, novel oxidative approaches, employing nonclassical solvents such as ionic liquids (ILs) and deep eutectic solvents (DESs), have been developed as greener and more sustainable biocatalytic synthetic pathways. This minireview aims highlights the recent advances made in the biocatalytic synthesis of enantioenriched sulfoxides by employing such unconventional approaches.

## Graphical abstract

**Going greener**: Enantioenriched sulfoxides can be synthesized by exploiting the new reductive enzymes Msr and Dms or using classical oxidative enzymes in the presence of unconventional solvents in a greener and more sustainable way.

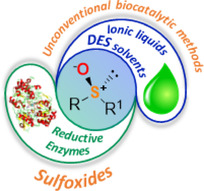

## 1 Introduction

**Figure 1:**
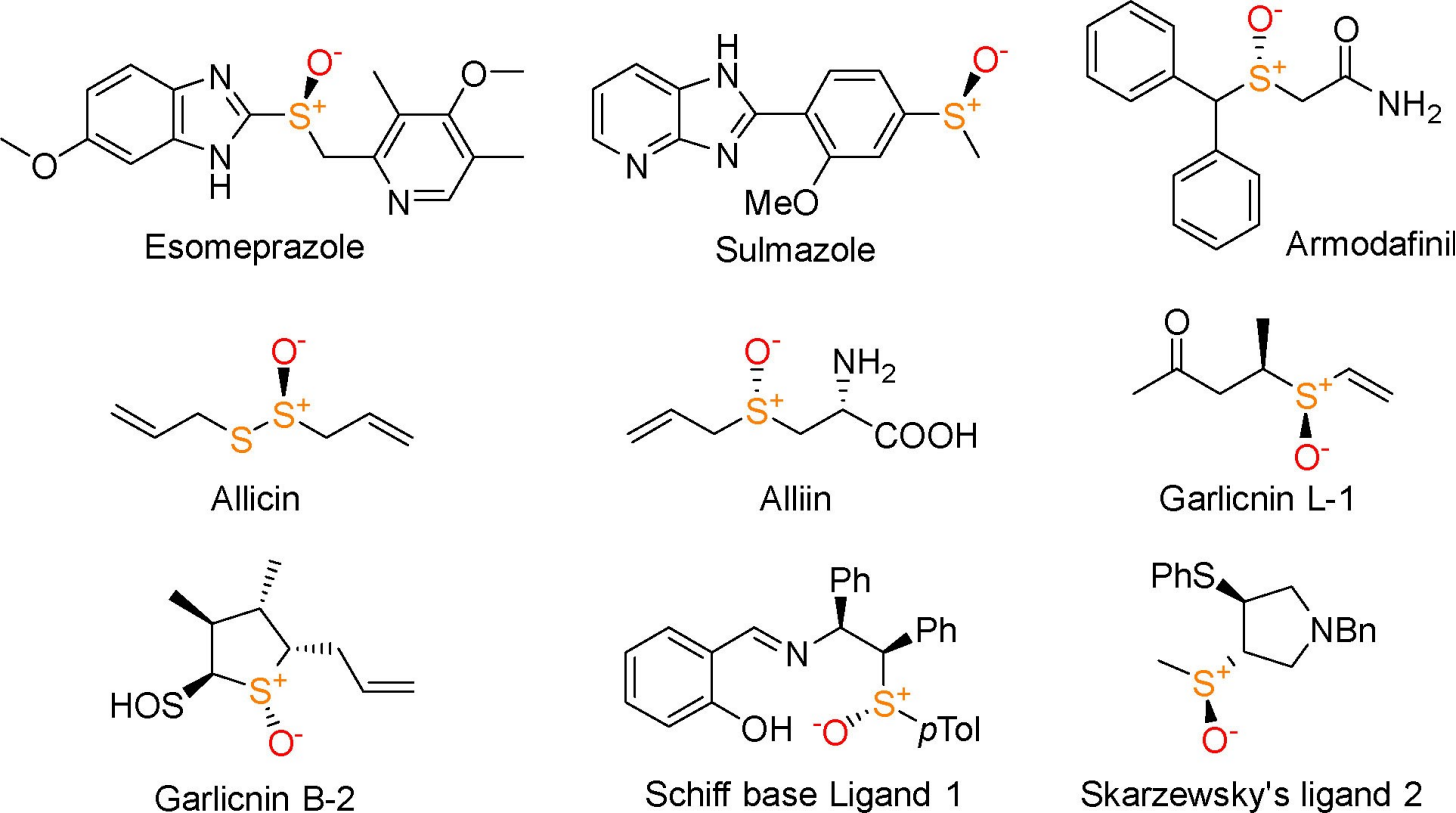
Natural sulfoxide‐ and sulfoxide‐containing drugs and ligands.

Sulfoxides are a wide class of organic compounds containing sulfur and oxygen with general formula RSOR′, where the R and R′ are carbon groups (or hydrogen) and the oxygen is directly bound to the sulfur atom.^[1]^ The unique peculiarity of sulfoxides is represented by the fact that the sulfur atom is a stereogenic centre when R≠R^1^ and it assumes a tetrahedral sp^3^ hybridization with a lone pair occupying one of the sp^3^ orbitals while the oxygen atom forms a d‐π bonding with sulfur. Generally, chiral sulfoxides are conformationally stable at ambient temperature and racemise only under harsh conditions. Thanks to their properties, enantiopure sulfoxides have attracted much attention in chemistry as they are found in many natural products and pharmaceutical agents such as the natural antibacterial garlic components^[2]^ allicin, ajoene and garlicnins B‐2 and L‐1^[3]^ as well as in the commercial drugs esomeprazole,^[4]^ (+)‐sulmazole^[5]^ and armodafinil.^[6]^ Enantiopure sulfoxides are also used in chemistry as chiral ligands for asymmetric organic syntheses, such as the Schiff base ligand **1** or the Skarzewsky's ligand **2** (Figure 1).^[7]^ The synthesis of organic compounds containing an enantiopure sulfoxide moiety is an attractive and challenging field in organic chemistry.[^7^, ^8^, ^9^]

When considering the synthesis of sulfoxides, research is naturally prone to approach the challenge by looking for an oxidative pathway as sulfoxides are the first oxide of their sulfide counterpart and, therefore, oxidative routes have been the first synthetic choice for many years.[^7^, ^8^, ^9^] The asymmetric synthesis of optically active sulfoxides has relied mainly on the use of chiral auxiliaries or metal catalysts.[^8^, ^9^, ^10^] In the last decades, due to the progresses made in the field of gene cloning, DNA sequencing and engineering and protein expression, a number of biocatalytic methods to construct enantiopure sulfoxides has been reported in literature as well. The vast majority of these enzymatic approaches exploit oxidative enzymes like monooxygenases, peroxidases or cytochromes P450,[^9^, ^11^, ^12^, ^13^, ^14^, ^15^] which convert achiral sulfides into enantioenriched sulfoxides. More recently, the use of reductive enzymes, able to catalyse the stereoselective reduction of racemic sulfoxides, has emerged as a valid alternative to standard oxidative biocatalytic pathways. This minireview aims at highlighting the recent advances made in the last decade in the synthesis of optically active sulfoxides by unconventional biocatalytic methods. While a number of reviews on the synthesis of sulfoxides using oxidising enzymes has been recently reported,[^9^, ^14^, ^15^] this review will focus mainly on the new reductive enzymatic pathways. In addition, the use of unconventional solvents, such as ionic liquids and deep eutectic solvents, in the biocatalytic synthesis of sulfoxides will be discussed, to highlight the advances made in the development of greener and more sustainable synthetic processes.

## 2 Biocatalytic Reduction of Chiral Sulfoxides

Enantiopure sulfoxides can be obtained by reductive biocatalytic mechanisms where reductive enzymes catalyse the kinetic resolution of sulfoxide racemates by selectively reducing one of the two enantiomers into the corresponding sulfide. Unlike the large pool of oxidative enzymes from which the researcher can chose from, the range of reductive enzymes is still rather small and, indeed, currently limited to only two classes: the methionine sulfoxide reductases (Msr) and the dimethyl sulfoxide (DMSO) reductases (DmsABC).

### 2.1 Methionine sulfoxide reductases

**Scheme 1:**
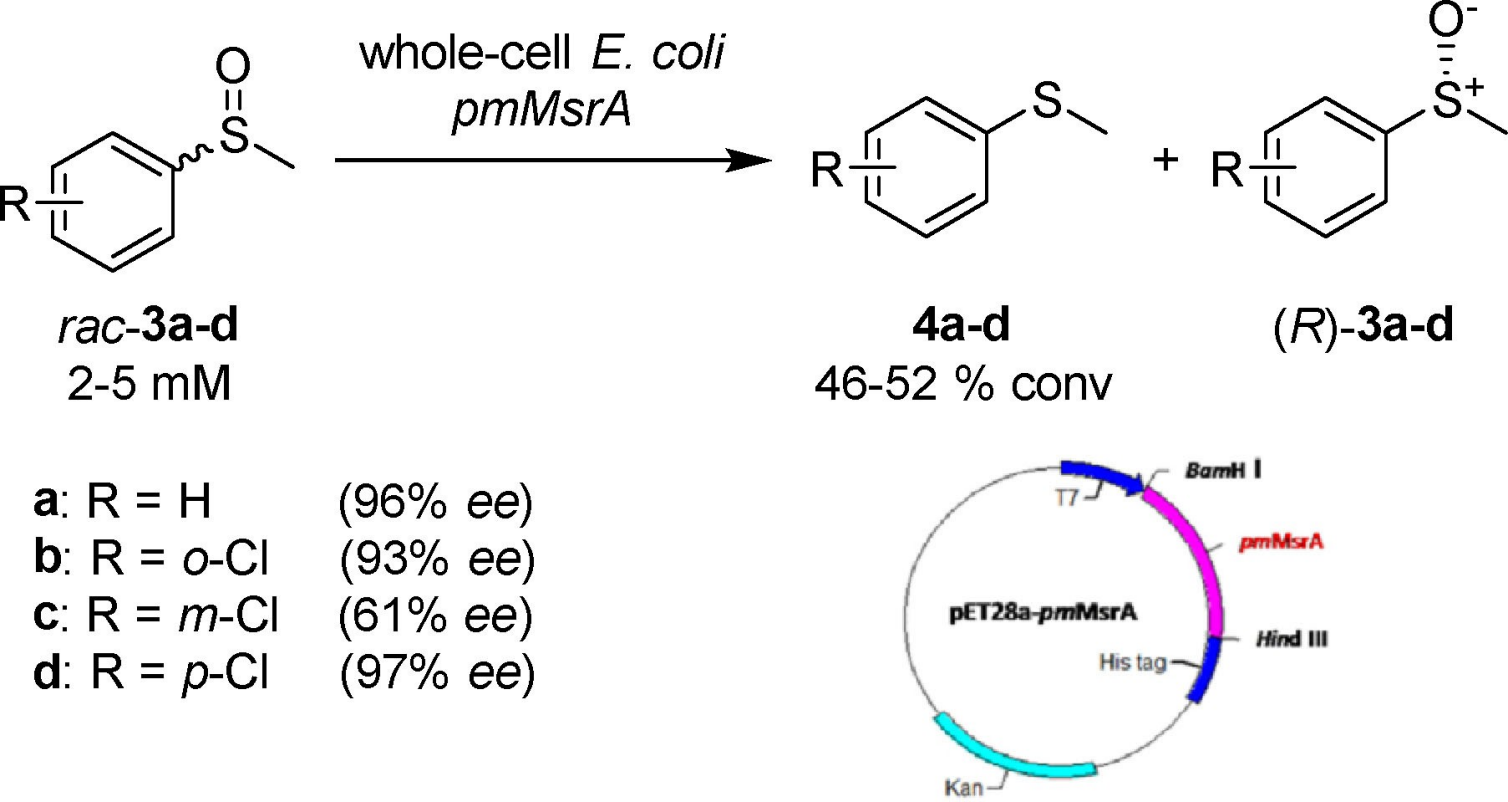
Synthesis of sulfoxides by using a whole *E. coli* cell system overexpressing pmMsrA.

The fundamental biochemical role of Msr enzymes is the ability of restoring the functionality of damaged proteins containing methionine sulfoxides. In cells, the oxidation of the amino acid methionine (Met) by reactive oxygen species (ROS) occurs frequently during cellular metabolism resulting in the formation of a diastereomeric mixture of Met‐(*S*)*‐*sulfoxide [Met‐*S*‐(O)] and Met‐(*R*)‐sulfoxide [Met‐*R*‐(O)].^[16]^ The original functionality of proteins containing Met‐*S*‐(O) and Met‐*R*‐(O) is restored by two subfamilies of Msr enzymes, the methionine sulfoxide reductases A (Msr‐A) and the methionine sulfoxide reductases B (Msr‐B). These enzymes are capable of reducing the Met‐*S*‐(O) and Met‐*R*‐(O) respectively back to the original amino acid Met.[^17^, ^18^] Following this natural biochemical reactivity, Msrs have been investigated as biocatalysts to perform the kinetic resolution of exogenous racemic sulfoxide substrates. Despite their activity being known for decades,^[19]^ only in 1992 Broth et al. first sequenced and expressed a recombinant Msr enzyme after cloning the gene from *Escherichia coli*.^[20]^ In 1996, the same group reported the cloning, sequencing and expression of the mammalian homologue of *E. coli* MsrA and showed that this enzyme was active on both natural and synthetic substrates^[21]^ and able to reduce a variety of sulfoxide containing compounds, including (*S*)‐(−)‐methyl *p*‐tolyl sulfoxide. However, concrete advances in the kinetic resolution of racemic sulfoxides using Msr enzymes truly happened only in the last five years as the progress and development of more advanced chemical biology techniques allowed research groups to re‐evaluate this class of enzymes as biocatalysts. Chen and co‐workers observed that a strain of *Pseudomonas monteilii* CCTCC M2013683 was capable of synthesising chiral sulfoxides with 99 % *ee*.^[22]^ Later, the same authors reported the cloning and expression of the MsrA gene from *P. monteilii* CCTCC M2013683 (pmMsrA).^[23]^ In order to assess the ability of pmMsrA to furnish optically pure sulfoxides, the recombinant protein was expressed in *E. coli*, harvested in the resting phase and subsequently subjected to an activity assay using *rac*‐**3**. After 24 hours, this whole cell system led to the formation of **4** with 51 % conversion, leaving (*R*)‐**3** unreacted >99 % *ee*. Further investigation of pmMsrA revealed that the system could tolerate substrate concentrations up to 5 mM with an optimal cell density of 40 g_cdw_ L^−1^ yielding 46 % (*R*)‐**3** after 16 h reaction and maintaining an excellent 96 % *ee*. The biocatalyst proved to tolerate halogen substitutions on the aromatic ring of **3** retaining good‐to‐excellent *ee* values and conversions (Scheme 1).

**Scheme 2:**
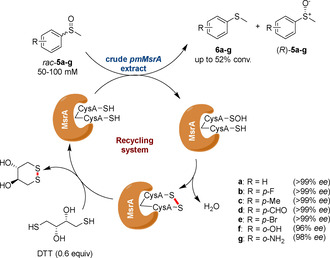
Conversions of racemic sulfoxides using the crude pmMsrA‐DTT system.

In 2017 Minetti et al. reported a recombinant mammalian MsrA showing a similar level of activity and enantioselectivity to Chen's biocatalyst.^[24]^ Noteworthy, a highly stereoselective kinetic resolution of racemic alkyl‐aryl‐sulfoxides using purified MsrA regenerated by the cheap and widely available dithiothreitol (DTT) was achieved, demonstrating that isolated Msrs are equally efficient for the synthesis of chiral sulfoxides. Following this work, Chen and Yang developed a crude pmMsrA‐DTT system that could tolerate substrate concentrations up to 200 mM (37 g L^−1^),^[25]^ and was found to reduce 50 and 200 mM *rac*‐**5** in 30 minutes and 4 hours, respectively, retaining >99 % *ee* in both cases (Scheme 2). Theoretically, in this system, only 0.5 equivalents of DTT should be necessary, as (*R*)‐**5** is only half of the overall amount of *rac*‐**5**. However, it was shown that moving from 0.5 to 0.6 equivalents of DTT was necessary as it was hypothesised that other cellular components in the crude enzyme extract may react and sequester it from the regeneration of pmMsrA. The use of whole cell pmMsrA combined with DTT was also evaluated to further simplify the system, leading however, to a lower optical purity after the same 2 hour time point (93 % *ee*). Chen's pmMsrA‐DTT system was found to be active on several sulfoxides *rac*‐**5 a**–**g** with excellent conversions and *ee* (Scheme 2).

**Scheme 3:**
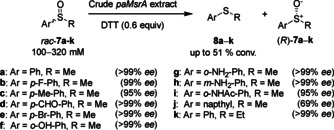
Conversion of racemic sulfoxides by using the crude paMsrA‐DTT system.

In 2019, Chen's group reported a homologue of pmMsrA enzyme from *Pseudomonas alcaliphila*, the biocatalyst paMsrA, that could tolerate substrate loadings up to 320 mM (45 g L^−1^).^[26]^ Four homologues of pmMsrA, namely pcMsrA, pfMsrA, paMsrA and vhMsrA sharing 60–90 % sequence identities, were identified and all showed similar biocatalytic activity to the parent enzyme in reducing *rac*‐**7 a**–**k** (Scheme 3). The crude paMsrA‐DTT system exhibited much better catalytic activity and stereoselectivity than other homologues.

**Scheme 4:**
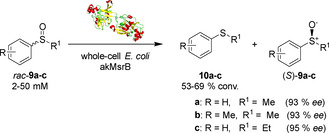
Conversion of racemic sulfoxides by using a whole *E. coli* cell system overexpressing akMsrB.

MsrAs are selective biocatalysts able to afford the (*R*)*‐*sulfoxide enantiomer. On the other hand, MsrB enzymes show opposite stereoselectivity and prove to be valid biocatalysts for the reduction Met‐*R*‐(O). However, MsrB enzymes have shown to be far less active and to have a much higher substrate specificity than MsrAs, thus limiting their use in the synthesis of (*S*)*‐*sulfoxides.^[27]^ In 2020, Chen and co‐workers reported the first example of kinetic resolution of alkyl aryl sulfoxides using whole cell akMsrB from *Acidovorax* sp. KKS102.^[28]^ The biocatalyst akMsrB was found among a pool of six enzymes that shared 55–92 % sequence identity out of which pmMsrB was able to convert the *R* enantiomers of sulfoxides *rac*‐**9 a**–**c** into the corresponding sulfides **10 a**–**c** yielding (*S*)*‐* **9 a**–**c** with >90 % *ee* (Scheme 4). Unfortunately, when the same biocatalytic transformation was attempted with the purified enzyme, all activity was lost.

The main drawback with the use of Msr catalysed processes is that the yield of the enantiopure sulfoxide can only have max. 50 %. Even if the sulfoxide/sulfide products of these biotransformations can be easily separated by chromatography, this may represent a major limitation at industrial level.

**Scheme 5:**
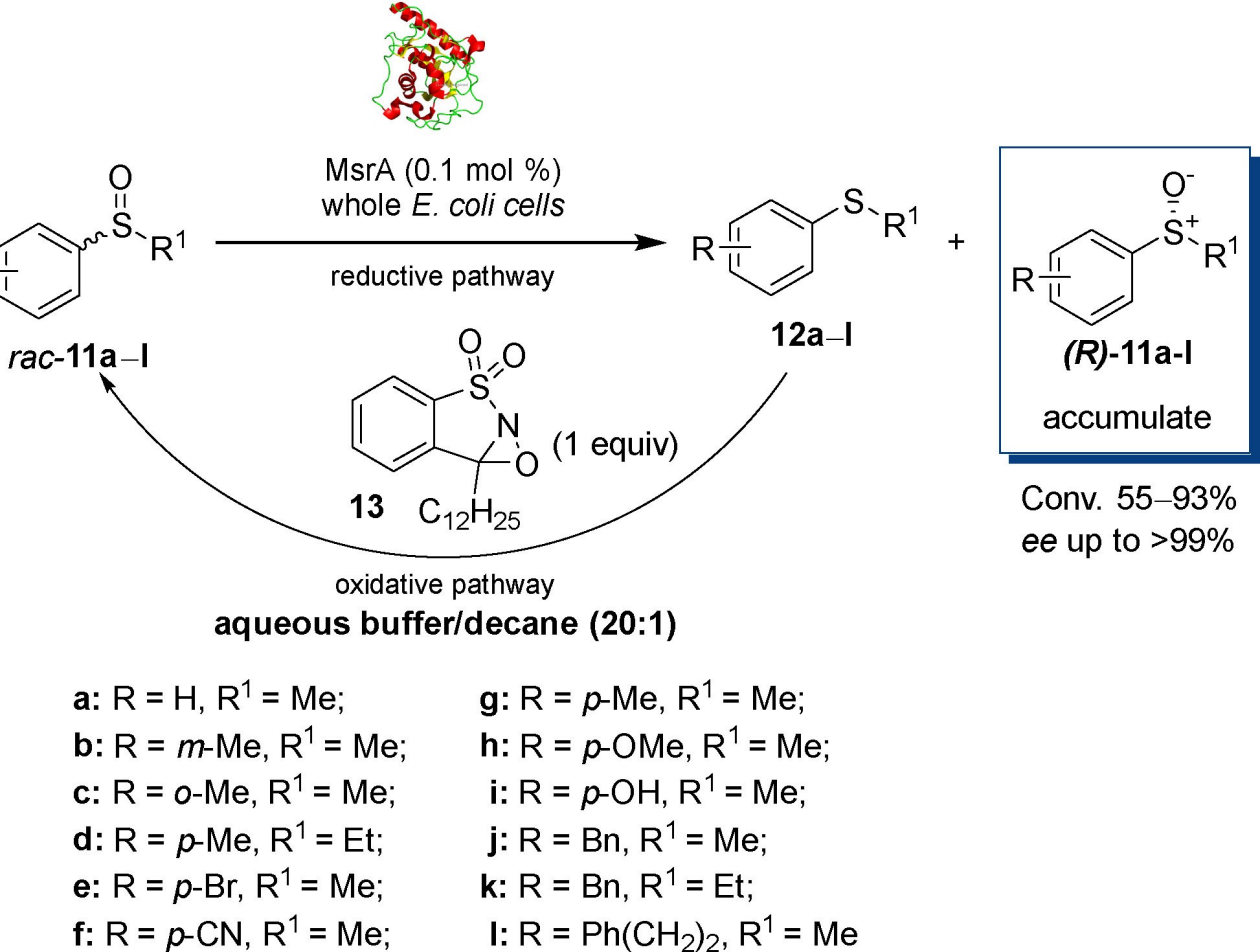
Deracemisation of racemic sulfoxides in a biphasic aqueous buffer/decane system by using whole cell MsrA and oxaziridine **13**.

In 2018, Míšek's group reported a chemo‐enzymatic dynamic deracemisation of sulfoxides *rac*‐**11 a**–**l** using whole cell *E. coli* overexpressing MsrA combined with an oxaziridine‐type oxidant **13** in a biphasic system.^[29]^ The rationale behind the use of a biphasic system was that once the stereoselective reduction of *rac*‐**11** happened in the aqueous buffer, the oxidation of **12** using the peroxide **13** could occur in the decane phase (5 % *v/v*), without inactivating the biocatalytic system. Sulfoxides *rac*‐**11 a**–**l** were converted into (*R*)*‐* **11 a**–**l** with excellent *ee* (>99 %) and moderate‐to‐good conversions (55–93 %; Scheme 5).The deracemization of the anti‐inflammatory drug sulindac was also carried out using this method, leading to *R* enantiomer with 93 % *ee* and 75 % conversion. The use of different oxidants (aliphatic peroxides) proved to be detrimental for the biotransformation.

### 2.2 Dimethyl sulfoxide reductases

DmsABC is a membrane bound enzyme made of three non‐identical subunits, namely a hydrophilic catalytic subunit (DmsA) that bears a pterin molybdenum cofactor, another hydrophilic subunit (DmsB) that contains four cysteine groups individually bonded to four [4Fe–4S] clusters and, finally, a hydrophobic subunit (DmsC) that anchors the protein to the cell membrane.^[30]^ This molybdoenzyme catalyses electron transfers from nitric oxide reductase (menaquinol) to a variety of N‐oxides and S‐oxides, including DMSO, during bacterial anaerobic cell respiration. Despite the ability of cells to reduce DMSO to dimethyl sulfide (DMS) being observed since the late 1950s,^[31]^ this catalytic activity was wrongly attributed to the presence of non‐specific reductases. In 1985, finally, the existence of DmsABC was established by Bilous and Weiner who managed to anaerobically grow *E. coli* cells solely on DMSO as the terminal electron acceptor.^[32]^ The authors also confirmed that the catalytic activity of this new enzyme was due to the presence of a molybdenum cofactor as it was observed that the exposure of the enzyme to sodium tungstate (Na_2_WO_4_) inhibited the catalytic activity just like previously observed in other molybdoenzymes, trimethylamine‐*N*‐oxide and nitrate reductases.

**Scheme 6:**
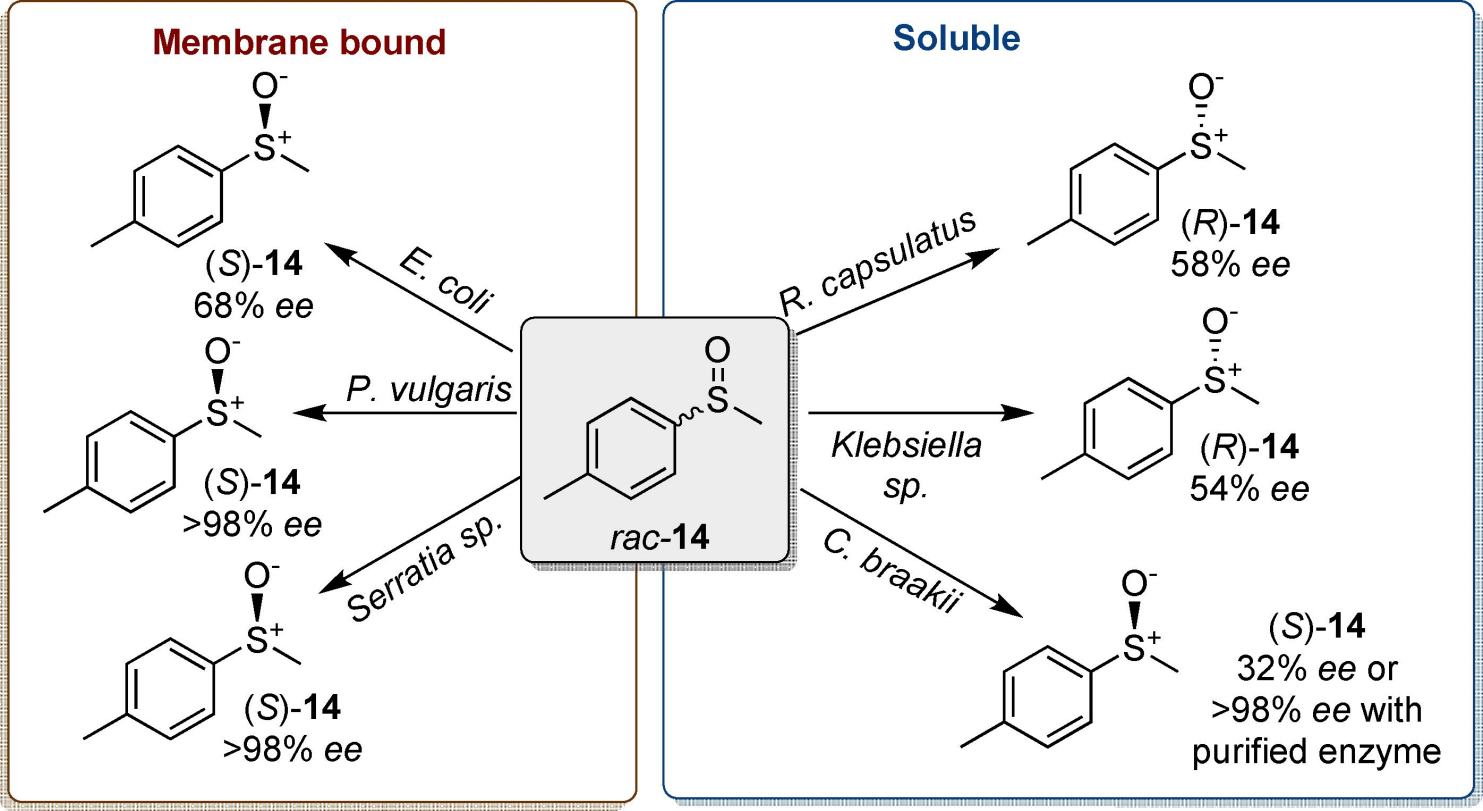
Selectivity of DmsABC found in different anaerobic bacteria species.

In 1994 Abo et al. first described the use of DmsABC from *Rhodobacter sphaeroides* f. s. *denitrificans* for the kinetic resolution of the non‐endogenous methyl phenyl sulfoxide, obtaining (*R*)‐methyl phenyl sulfoxide in 42 % yield and 97 % *ee*.^[33]^ Following this initial work, the substrate scope of this biotransformation was further expanded to a variety of alkyl‐aryl‐sulfoxides.[^34^, ^35^, ^36^] In 2004, Luckarift et al. described the kinetic resolution of chiral sulfoxides with opposite stereoselectivities depending on the species of anaerobic bacteria used^[37]^ (Scheme 6). A total of seven organisms used as whole cell biocatalysts were exposed to 6.5 mM (1 g L^−1^) *rac*‐**14** and it was found that *E. coli*, *Proteur vulgaris*, *Serratia* sp., *Citrobacter braakii* and *Halobacterium halobium* selectively reduced the *R* enantiomer while *Rhodobacter capsulatus* and *Klebsiella* sp. reduced the *S* enantiomer. These whole cell enzymes were found to have moderate to excellent optical purities (36 to >98 % *ee*). Upon expansion of the substrate scope, the group observed that the *R* enantiopreference was often exhibited by membrane‐bound reductases whereas soluble enzymes favoured the reduction of the *S* enantiomer with the exception of *C. braakii*. The biocatalytic activity of the latter as isolated enzyme was also investigated affording (*S*)‐**14** in >98 % *ee*.

**Scheme 7:**
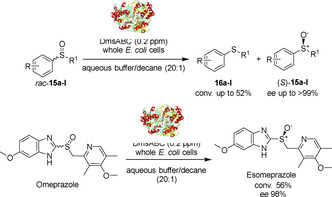
Kinetic resolution of alkyl aryl sulfoxides by using DmsABC.

In an attempt to obtain a system with opposite stereoselectivity to MsrA biocatalysts, in 2019 Míšek and co‐workers screened a panel of MsrB enzymes. Even if the study did not lead to the desired outcome, the authors observed that resting phase whole cell *E. coli* still possessed (*R*)*‐*sulfoxide reducing activity; this catalytic activity was then assigned to dimethyl sulfoxide reductase (DmsABC).^[38]^ Later, the authors reported the kinetic resolution of various racemic sulfoxides *rac*‐**15 a**–**l** using whole cell *E. coli* DmsABC^[39]^ (Scheme 7). Similar to their previous MsrA deracemisation method, this new system operated in aqueous buffer/decane biphasic conditions and showed high enantioselectivity (up to >99 % *ee*) and conversions (up to 52 %). Interestingly, the substrate scope of DmsABC resulted to be slightly wider than that of MrsA. Finally, omeprazole was converted to esomeprazole with 56 % conversion and 98 % *ee*. In this case, the biotransformation was carried out at pH up to 9 and without organic co‐solvent.

One of the issues encountered with purified enzymes is that external reductants and expensive cofactors are usually needed to reactivate the enzyme after a turnover.

**Scheme 8:**
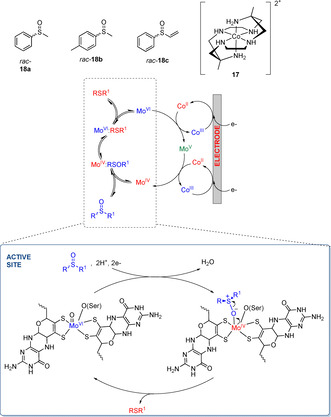
Mechanism of the electroenzymatic kinetic resolution of *rac*‐**18 a**–**c**.

In 2015, Bernhardt et al. reported an electrochemically mediated kinetic resolution of racemic alkyl aryl sulfoxides using purified DmsABC from *R. capsulatus* in combination with the hexaaminecobalt coordination compound **17**. The latter was employed to transfer electrons from an electrode to the reductase, in turn allowing the continuous regeneration of the enzyme.^[40]^ (Scheme 8). Sulfoxides (*R*)‐**18 a**–**c** were obtained with 71 to >99 % *ee* after exposing DmsABC to 0.7 g L^−1^ of racemic *rac*‐**18**. The lower enantioselectivity observed with *rac*‐**18 c** was associated to the bulkier vinyl substituent that determined a poorer coordination to the Mo ion. The mechanism of regeneration of DmsABC proposed by the group is shown in Scheme 8. The molybdenum cofactor is part of a redox cycle that allows the metal ion to continuously reduce the sulfoxide to the corresponding sulfide as the cobalt complex to switch between its two oxidation states [Co(*trans*‐diammac)]^2+^ and [Co(*trans*‐diammac)]^3+^.

**Scheme 9:**
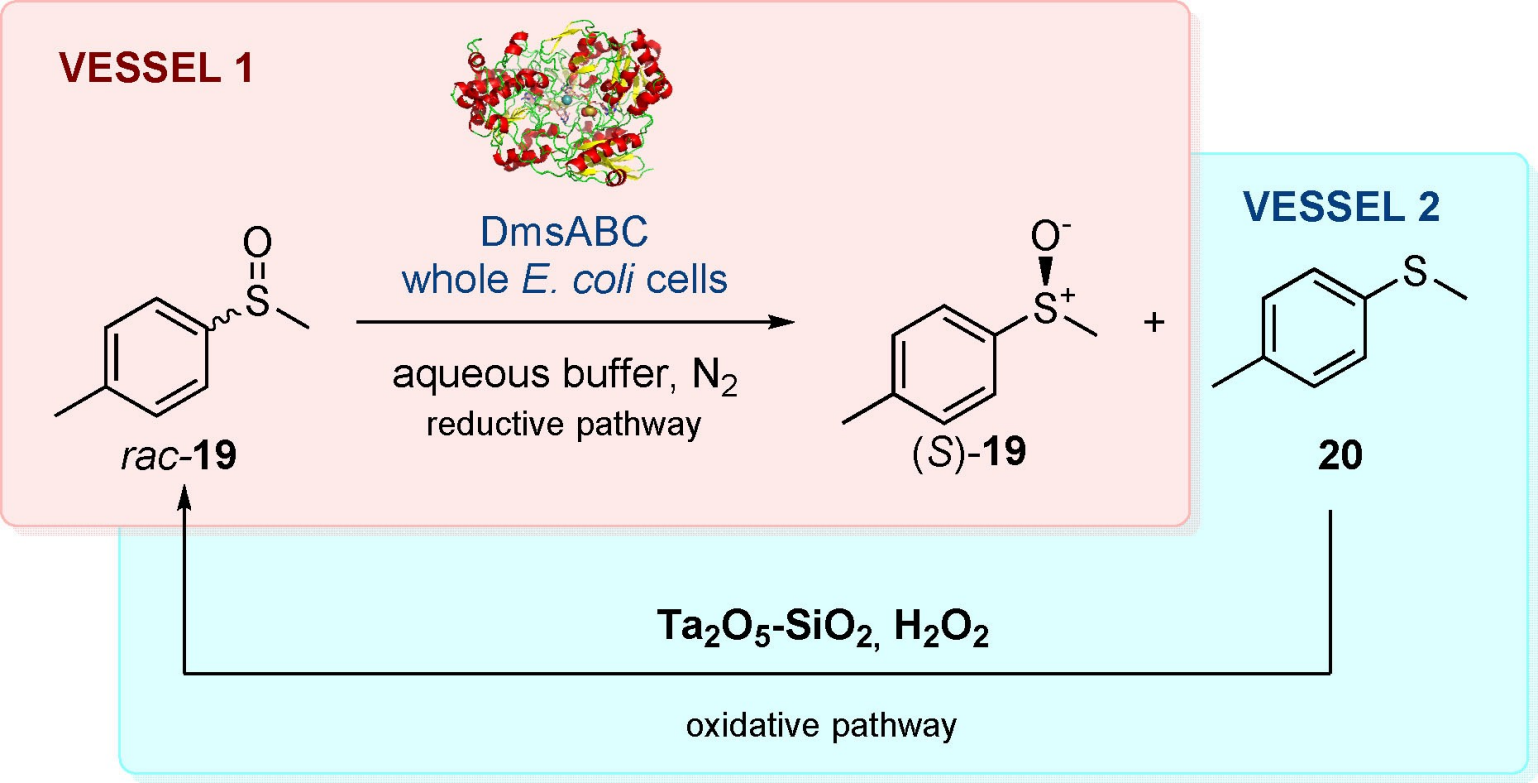
Chemoenzymatic deracemisation of *rac*‐**19** by using whole‐cell *E. coli* DmsABC.

In 2012, Parvulescu et al. combined biocatalysis with an inorganic metal catalyst describing a two‐step deracemization process with whole cell *E. coli* DmsABC and Ta_2_O_5_‐SiO_2_. The system was used for the synthesis of (*S*)‐**19** with 56 % conversion and 97 % *ee*.^[41]^ Whole cell DmsABC from different *E. coli* strains (ATTC11303, top 10, dalfa5HL and Mac1) were initially screened by the authors and DmsABC from *E. coli* ATTC11303 showed the highest stereoselectivity for (*S*)‐**19** in 38 % conversion and 62 % *ee*. The authors then combined the reductive kinetic resolution with an oxidation step, using silica incorporated tantalum Ta_2_O_5_−SiO_2_ (15 wt %) as oxidant together with 1 equiv. of H_2_O_2_. Interestingly, the oxidation was performed in the ionic liquid (IL) [BMIM][NTf_2_]. However, given the incompatibility of the IL and the enzymatic buffer aqueous phase, the deracemisation of *rac*‐**19** was carried out in two separate vessels. First, the racemate was subjected to three cycles of enantioselective reduction by DmsABC (prepared fresh for each cycle), which yielded (*S*)‐**19** in 49 % conversion and 97 % *ee*. Then, after extraction and purification of the products, **20** was re‐oxidised to *rac*‐**19** using the Ta_2_O_5_−SiO_2_/H_2_O_2_ system in the IL. Finally, the freshly prepared *rac*‐**19** was once again exposed to three cycles of enantioselective reduction by DmsABC to afford (*S*)‐**19** in 97 % *ee*. These subsequent steps led to a deracemization process with a total 56 % conversion, which showed a 20 % enhancement compared to the bioreduction only (Scheme 9).

Overall, DmsABC enzymes have proven to provide valid alternative methods for the synthesis of asymmetric sulfoxides. Unlike Msrs, DmsABCs have however mostly been used as whole cell biocatalysts due to the membrane‐bound nature of these molybdoenzymes, which resulted on far lower substrate loadings (only up to 1 g L^−1^) to avoid toxicity to the cells. In addition, most of the DmsABC catalysed biotransformations must be carried out under inert atmosphere (N_2_ or Ar) to ensure anaerobic growth of the bacteria and upregulation of the reductase. The use of transition metals in order to successfully synthesise chiral sulfoxides is very promising but greener and more efficient ways to both regenerate the catalyst and perform *in situ* oxidations without deactivating the enzymes will be needed in the future.

## 3 Biocatalytic Synthesis of Sulfoxides in Unconventional Media

Although biocatalysis is considered a green methodology, organic solvents are often required in biotransformations as co‐solvents and additives to favour and increase the solubility of unnatural substrates in buffer solutions. Organic petroleum‐based solvents can be toxic, hazardous, non‐renewable and in some instances require expensive waste processing. Therefore, researchers have investigated the possibility to replace these co‐solvents with greener and more sustainable alternatives. In this section, the biocatalytic synthesis of sulfoxides in unconventional media, namely ionic liquids (ILs) and their analogues deep eutectic solvents (DESs), is described. It must be emphasised that the use of ILs and DESs in biocatalysis offers several advantages in terms of green chemistry, other than the simple avoidance of toxic and hazardous conventional organic co‐solvents. In fact, both ILs and DESs may improve the stability of the enzymes, due to the interactions between their ionic charges with those of the biocatalysts, in turn leading to an increase in the yield and the enantioselectivity of the whole biocatalytic processes.^[42]^ As the use of reductive enzymes in the synthesis of sulfoxides is a recent outcome, all the methods employing unconventional IL/DES solvents concern the oxidation of sulfide substrates.

### 3.1 Ionic liquids (ILs)

Discovered in the 20th century, ILs are organic salts that are liquid at temperatures below 100 °C. ILs have an number of unique properties including non‐volatility, non‐flammability, negligible vapour pressure, excellent chemical and thermal stabilities, all of which have made them an attractive green alternative to traditional organic solvents.^[43]^ One of the earliest ILs described in literature was ethylammonium nitrate, firstly reported in 1914 by Walden and co‐workers who found that this salt had a melting point of 12 °C.^[44]^ Since then, a number of other ILs with unique properties, including high polarity and hydrophobicity, have been identified, making them exploitable in a wide range of potential chemical applications.^[45]^ The use of ILs in biocatalysis is a relatively recent occurrence driven both by the increasing importance placed upon green chemistry and the need to improve enzyme turnover rate. In the 1980s, Zaks and Klibanov demonstrated how the enzyme activity in organic solvents is dependent upon the hydrophilicity of the solvent used: the more hydrophilic the solvent is, the lower enzyme activity is observed.[^46^, ^47^] The hydrophobic nature of ILs, their immiscibility with water as well as their ability to increase the enzymes stability and selectivity make them ideal greener solvent exploitable in biocatalysis.^[42]^

The first enzymatic reaction using ILs appeared in 2000, involving whole‐cell *Rhodococcus* R312 catalysed hydrolysis of 1,3‐dicyanobenzene using a biphasic buffer/1‐butyl‐3‐methylimidazolium hexafluorophosphate ([BMIM]PF_6_) system exploiting nitrilase activity.^[48]^ After this initial report, other enzymes, including lipases,[^49^, ^50^] proteases^[51]^ and glycosidases^[52]^ have been used in the presence of ILs leading to highly regioselective and enantioselective biotransformations.

**Scheme 10:**
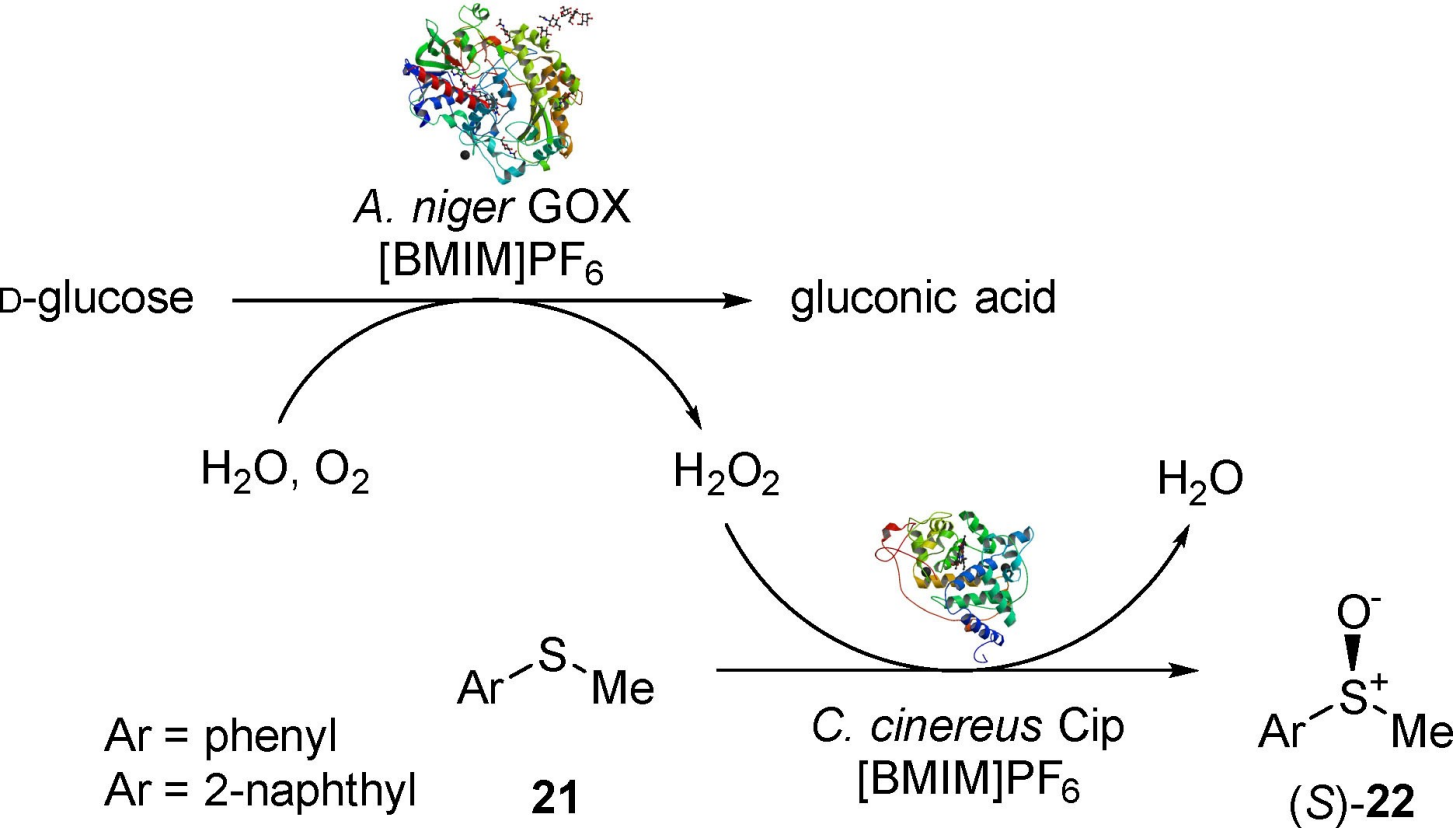
GOX‐/Cip‐catalysed enantioselective sulfoxidation of aryl sulfoxides in IL [BMIM]PF_6_.

The first report exploring the oxidation of sulfides in ionic liquids was published in 2003 by Okrasa et al.^[53]^ Hydrogen peroxide was produced *in‐situ* by glucose oxidase (GOX) from *Aspergilius niger* and used by peroxidase from *Coprinus cinereus* (Cip) to catalyse the oxidation of arylmethyl sulfide substrates **21** into the corresponding sulfoxides (*S*)‐**22**. This bi‐enzymatic biotransformation was carried out in the ionic liquid 1‐butyl‐3‐methylimidazolium hexafluorophoshate ([BMIM]PF_6_) in the presence of different concentrations (1–10 %) of water (Scheme 10). The *in‐situ* production of H_2_O_2_ proved to be crucial for this reaction, as the direct oxidation of substrates through slow addition of the peroxide to the IL suspension of Cip was unsuccessful. Hydrogen peroxide formation was affected by the concentration of water added to the IL suspension, with 5 % (*v/v*) water content sufficient for the optimum activity of GOX, and 10 % (*v/v*) water content providing the ideal conditions for both enzymes. Consequently, [BMIM]PF_6_ with 10 % (*v/v*) water content was employed as optimal reaction medium. Sulfoxides (*S*)‐**22** were obtained with 32–36 % conversion and 68–91 % *ee*. It is noteworthy that both enzymes had high operational stability in [BMIM]PF_6_ and that after 32 hours both the sulfoxide and remaining sulfide could be extracted and the IL re‐used in a new cycle with similar stereoselectivity and conversion rates.

**Scheme 11:**
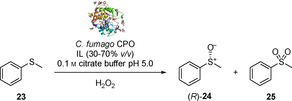
CPO‐catalysed sulfoxidation of methyl phenyl sulfide **23** in ILs.

In 2006, Chiappe et al. investigated the viability of seven hydrophobic ILs as co‐solvents in the chloroperoxidase (CPO) catalysed sulfoxidation of methyl phenyl sulfide **23** to sulfoxide (*R*)‐**24** (Scheme 11).^[54]^ In [mmim][MeSO_4_], [Mor_11_][MeSO_4_] and [N_1112_OH][H_2_PO_4_], the CPO enzyme lost activity and only *rac*‐**24** could be formed. On the other hand, in [mmim][Me_2_PO_4_], [N_1112_OH][OAc] and [N_1112_OH][Citr] enantiomerically pure (*R*)‐**24** was obtained. In particular, in the presence of [mmim][Me_2_PO_4_], (*R*)‐**24** was obtained with 76 % conversion and excellent >99 % *ee*, while only 2 % of overoxidation product **25** was formed. By comparison conversion in pure buffer was 35 % of which 89 % sulfoxide and 11 % sulfone, with a sulfoxide *ee* of 97 %. These results clearly show the viability and the beneficial effect of ILs in biocatalysis as co‐solvents in replacement of traditional organic solvents for this system. The formation of racemic sulfoxide products in some cases was attributed to the fact that ILs led to a shifting of the reaction medium pH to >6.0 or below 2.7, thus probably inactivating the CPO.

Similarly, in 2008, Lichtenecker and Schmid investigated the activity of *Caldariomyces fumago* CPO in catalysing the sulfoxidation of **23** into sulfoxide **24** using a variety of ILs (20–40 % *v/v*) as a co‐solvent in the presence of acetate buffer.^[55]^ ILs were formed from 1‐butyl‐3‐methylimidazolium/1‐butyl‐3‐methylpyrrolidinium cations coupled to tosylate, chloride, trifluoromethylsulfonate, methylsulfate, nitrate, tetrafluoroborate or acetate anions. Hydrogen peroxide or *t*BuOOH were used as the oxidants, whilst control reactions using the most common conventional organic solvents *tert*‐butanol and acetone were run in parallel. Reaction mixtures with ILs containing chloride, tosylate and methylsulfate anions showed high conversion rates (up to 100 % with >81 % *ee*). When H_2_O_2_ was used as the oxidising agent, the conversion of **23** into **24** could be increased from 40 % (98 % *ee*) in pure acetate buffer to 80 % upon the addition of ILs (99 % *ee*). Whilst high conversion rates and *ee* values could be obtained, it is important to note there is much variation depending upon the composition and amount of the IL used as well as the type of oxidising agent employed. Generally, increasing the IL content from 20 to 40 % *v/v* led to increased conversion rates, while the choice of the appropriate IL and oxidant proved to affect the enantiomeric excess of the reaction products.

**Scheme 12:**
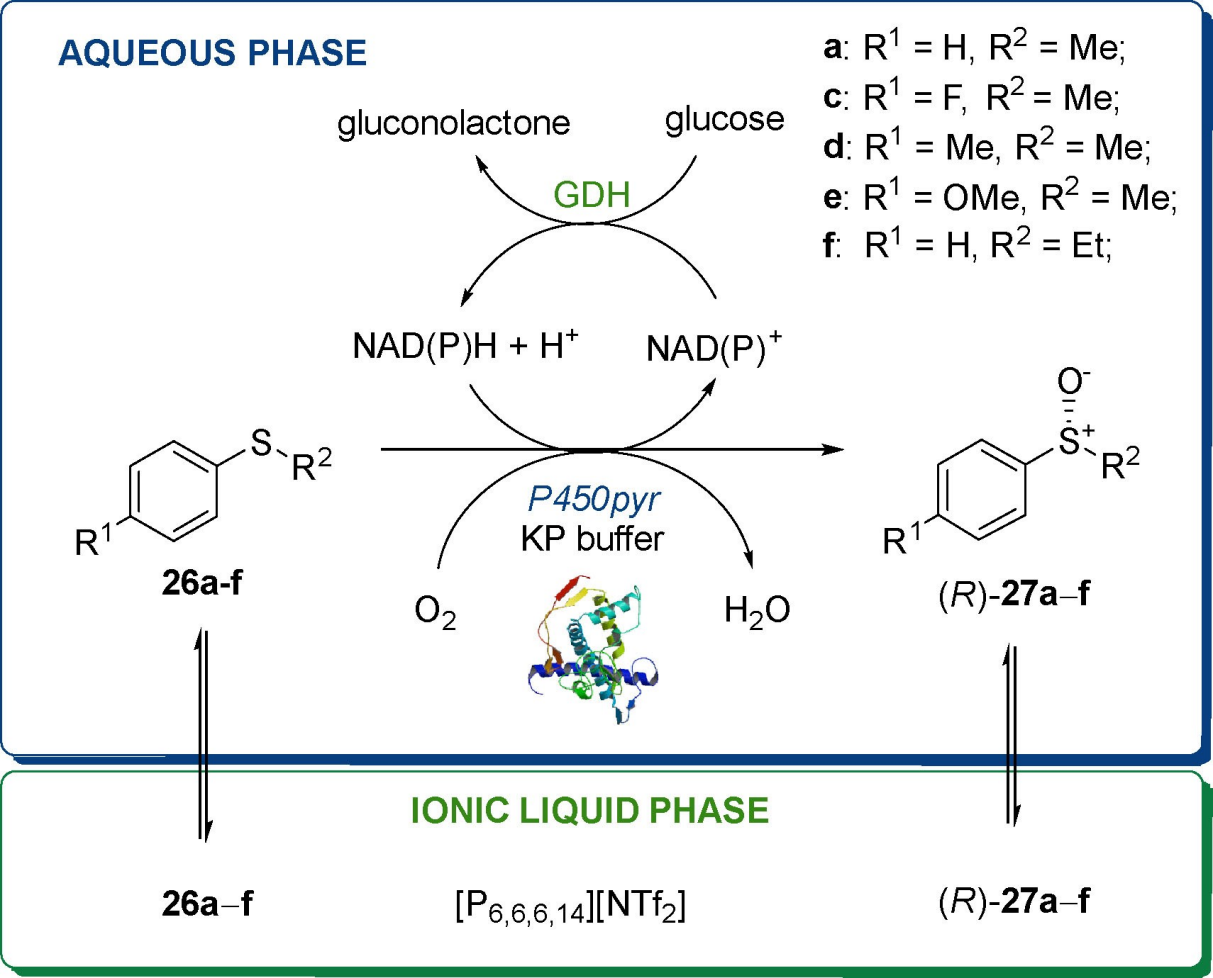
Asymmetric sulfoxidations with P450pyr in a KP Buffer/IL system.

More recently, in 2014, Gao et al. reported the use of an aqueous/IL biphasic system to enhance the activity of cytochromes P450‐catalysed asymmetric sulfoxidation of aryl sulfides.^[56]^ A novel P450pyr monooxygenase from *Spingomonas* sp. HXN‐200, the P450pyrI83H, was engineered to enhance its *R* enantioselectivity in biocatalysed oxidation reactions. P450pyrI83H was co‐expressed in *E. coli* with a glucose dehydrogenase (GDH), required to regenerate cofactor NADPH (Scheme 12). The asymmetric sulfoxidation of substrates **26 a**–**f** was first accomplished in KP buffer alone leading to the corresponding sulfoxides (*R*)‐**27 a**–**f** with good *ee* (35–86 %). The hydrophobic IL [P_6,6,6,14_][NTf_2_] was then selected on the basis of its excellent biocompatibility with *E. coli* and used in combination with the KP buffer in a biphasic KP buffer/[P_6,6,6,14_][NTf_2_] system. When the biocatalytic sulfoxidation was carried out in this biphasic medium, a significant enhancement in the enantioselectivity and conversion of the products was observed and compounds (*R*)‐**27 a**–**f** were obtained with good‐to excellent *ee* (62 to >99 %).

**Scheme 13:**
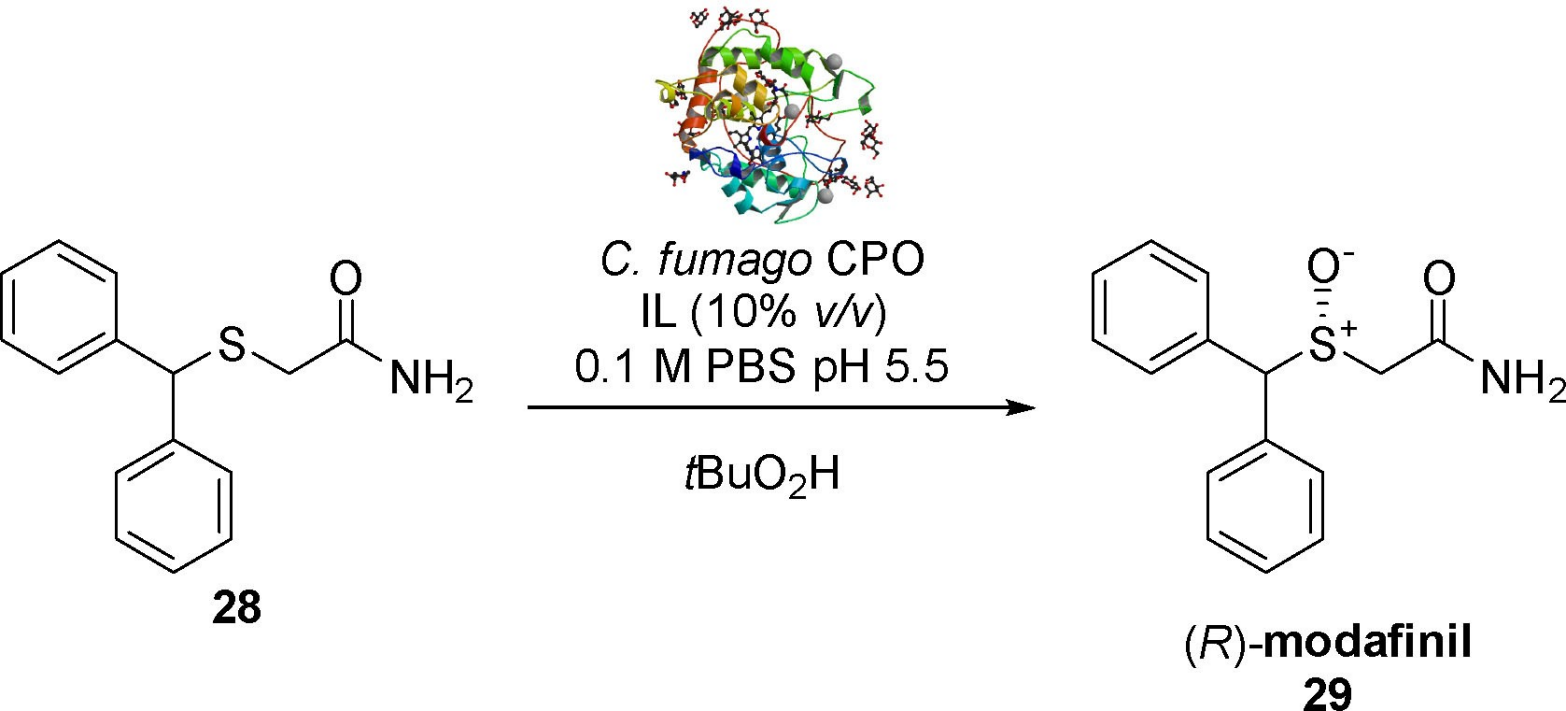
Synthesis of (*R*)‐modafinil **29** by using CPO from *C. fumago* in a biphasic aqueous buffer/IL.

In 2015, the sulfoxidation of **28** into (*R*)‐modafinil **29** using *C. fumago* CPO as isolated and purified enzyme in the presence of IL was described^[57]^ (Scheme 13). Unlike previous studies, *t*BuOOH was used as the oxidant. In aqueous phosphate buffer alone, (*R*)‐**29** was the predominant enantiomer formed with the best yield of 12 and 97 % *ee*. On the other hand, adding 1‐ethyl‐3‐methylimidazolium ([EMIM][Br]) IL 10 % (*v/v*) proved to be beneficial for the reaction, leading to an increase of the yield to 41 %, maintaining 97 % *ee*.

### 3.2 Deep eutectic solvents (DESs)

In 2001, Abbott et al. reported the synthesis of a number of new non‐aqueous solvents made from metal chlorides and composed of less reactive metal ions and cheaper quaternary ammonium salts that were fluid at ambient temperature.^[58]^ These solvents were easier to prepare than ILs, biodegradable and inert in water, making them a much more attractive alternative. It was later discovered that these solvents could be formed from a eutectic mixture of Lewis or Brønsted acids and bases, forming a fluid medium at <100 °C. As these liquid analogues showed different properties from ILs, they were deemed a new class of liquid in their own right and later termed deep eutectic solvents (DESs).^[59]^ The fluidity was attributed to the presence of large asymmetric ions in the DESs that generate low lattice energy, and produce low melting points.^[60]^ In the last 20 years, the field of deep eutectic solvents has burgeoned, many new DESs have been synthesised and classified into four different types^[61]^ and a great number of applications has been identified, including biocatalytic reactions. In particular, Type III DESs, which are from natural compounds containing a hydrogen bond donor and a quaternary ammonium salt such as organic acids, amino acids and natural sugars, are often described as being natural deep eutectic solvents (NADESs).^[62]^ The NADESs generally have a low melting point, are biodegradable, more sustainable and able to be produced from a wide range of naturally sourced compounds, thus turning to be a highly attractive green choice as reaction medium in biocatalysis. There are a number of comprehensive reviews published that delve deeper into DESs/NADESs and their multiple applications.[^60^, ^63^, ^64^, ^65^] The first use of DESs in biocatalysis was reported in 2008,^[66]^ followed by many other examples that demonstrated the successful employment of these solvents as reaction media for a number of different biocatalytic reactions.[^67^, ^68^, ^69^]

**Scheme 14:**
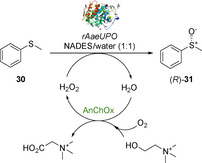
Bienzymatic sulfoxidation of **30** by using AnChOx and rAaeUPO.

Recently, the use of DESs in biocatalytic sulfoxidation has been reported by Li et al.^[70]^ A recombinant peroxygenase from *Agrocybe aegerita* (r*Aae*UPO) was used to catalyse the sulfoxidation of **30** in a biphasic water/NADES (urea‐ChCl) medium exploiting H_2_O_2_ produced *in situ* from the concomitant biocatalytic oxidation of water and ChCl by a choline oxidase from *Arthrobacter nicotianae* (*An*ChOx) water through a second biocatalytic oxidation (Scheme 14).

Urea‐ and propanediol‐containing NADESs gave the highest conversions at ∼45–60 %, whilst the biocatalytic reactions carried out with carbohydrate‐based NADESs showed the lowest conversions (<25 %). In all cases, enantiomerically pure (*R*)‐**31** was formed with >99 % *ee*. Additional experiments using a mono‐enzymatic system with *An*ChOx synthesising H_2_O_2_ *in situ*, led to the formation of **31** as racemate without enzymatic stereocontrol. This confirmed that r*Aae*UPO was required for stereoselective sulfoxidation. Interestingly, no reactivity in neat DES was observed, showing that a water content of at least 25 % is required for the enzymatic oxidation of ChCl and more in general for enzyme activity. The ChCl/urea NADES mixtures spontaneously adsorb up to 1 wt % water and therefore it was assumed that the NADES adsorbing the water molecules associated with the enzyme could be the cause for the absence of enzymatic activity.

## 4 Conclusions

In summary, this minireview highlights the new approaches developed for the biocatalytic synthesis of chiral sulfoxides. Although most of the biocatalytic methodologies reported to date rely on the oxidation of prochiral sulfides making use of monooxygenase, peroxidase or cytochromes P450, a new pool of reductive biocatalysts is emerging as valid alternative to access enantioenriched sulfoxides through reduction of the corresponding racemates. The development of Msr and DmsABC enzymes is an attracting research field since these biocatalysts have several features that are rather appealing to both academia and industry to access optically pure sulfoxides with high enantioselectivity and a wide substrate scope. Compared to the oxidative pathways, one of the advantages in using the reductive biocatalysts is that no external oxygen donors, such as explosive peroxides, are needed, making these methodologies particularly attractive from an industrial point of view. Even though the use of additives/auxiliaries is discouraged when developing green processes, the use of stoichiometric amounts of the non‐toxic and cheap DTT combined with crude MsrAs extracts allows for high substrate loadings still maintaining high conversions and optical purities. Also, the development of chemo‐enzymatic approaches combining DmsABC with electrochemical reactions or metal catalysts offers new opportunities to access enantioenriched sulfoxides with excellent conversions and *ee*. Finally, the possibility to use unconventional solvents in biocatalysis also represents an attractive research area. Ionic liquids and DESs proved to be suitable solvent/co‐solvent able to replace traditional organic solvents in the biocatalytic synthesis of sulfoxides leading to desired products often with improved yields and *ee* values. Even if, in terms of industrial applicability, the use of ILs and DESs still shows some limitations in terms of costs and substrate concentrations, it is undoubtful that they represent a promising and suitable alternative to classical organic solvents being cheaper, biodegradable and nontoxic.

## Conflict of interest

The authors declare no conflict of interest.

## Biographical Information

*Silvia Anselmi graduated in pharmaceutical chemistry from Queen Mary University of London in 2018, where her MSci project under the supervision of Dr. Lesley Howell concerned the design of small‐molecule inhibitors of the protein Mcl‐1. She was twice awarded the Drapers’ company undergraduate prize for academic achievement. In 2017, she also worked under the supervision of Dr. Stellios Arseniyadis on the development of a copper‐mediated asymmetric Michael addition/retro‐Dieckmann sequence. She is currently doing her PhD in D.C.’s group on the development of green biocatalytic methodologies for the synthesis of bioactive sulfoxide compounds*.

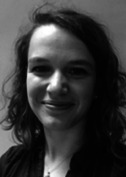

## Biographical Information

*Thomas S. Moody gained his Ph.D. in physical organic chemistry from The Queen's University of Belfast in 2001. He is an honorary Professor at Queen's University and has been part of their SAB since 2013. His work has earned him numerous accolades including the Irish Industrial Award for Chemistry (2017). Tom is a Vice President at Almac Sciences and Arran Chemical Company, where he is responsible for driving new technology processes from conception to commercial scale‐up across biocatalysis, flow chemistry, radiochemistry and custom synthesis. He has >20 years of academic and industry experience in chiral chemistry and biocatalysis*.

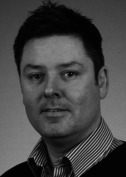

## Biographical Information

*Nandini Aggarwal obtained her BSc at King's College London in pharmacology in 2015, during which she joined the research group of Prof. Stuart Bevan at the Wolfson Centre for Age‐Related Diseases as an undergraduate research assistant. She worked for Boots UK in the pharmaceutical division before pursuing postgraduate study. She is presently completing a Master's in biomedical and molecular sciences research at King's College London and she is working on her Master's thesis in D.C.’s research group, researching sustainable approaches for the biocatalytic synthesis of chiral sulfoxides*.

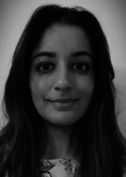

## Biographical Information

*Daniele Castagnolo obtained his PhD in pharmaceutical chemistry at the University of Siena in 2006 working under Prof. Maurizio Botta. He joined Prof. Johann Mulzer (University of Vienna) as a visiting PhD student, and carried out postdoctoral studies with Prof. Petri Pihko (Helsinki University of Technology), at the University of Siena and with Prof. Jonathan Clayden (University of Manchester). He started his independent research at Northumbria University Newcastle before moving to King's College London where he is currently Senior Lecturer in Pharmaceutical Chemistry. His research focuses on developing novel biocatalysed and chemo‐enzymatic reactions to synthesise drug‐like compounds and on discovering antimicrobial agents*.

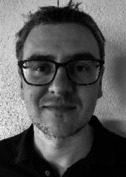

## References

[1a] H. Pellissier , Tetrahedron 2006, 62, 5559–5601;

[1b] I. Fernández , N. Khiar , Chem. Rev. 2003, 103, 3651–3706; 12964880 10.1021/cr990372u

[1c] M. C. Carreño , Chem. Rev. 1995, 95, 1717–1760.

[2] N. Martins , S. Petropoulos , I. C. Ferreira , Food Chem. 2016, 211, 41–50. 27283605 10.1016/j.foodchem.2016.05.029

[3] T. Nohara , Y. Fujiwara , T. Ikeda , K. Murakami , M. Ono , D. Nakano , J. Kinjo , Chem. Pharm. Bull. 2013, 61, 695–699. 10.1248/cpb.c13-00082 23812395

[4] M. J. Kendall , Aliment. Pharmacol. Ther. 2003, 17, 1–4. 10.1046/j.1365-2036.17.s1.1.x 12614298

[5] P. Barraclough , J. W. Black , D. Cambridge , D. Collard , D. Firmin , V. P. Gerskowitch , R. C. Glen , H. Giles , A. P. Hill , J. Med. Chem. 1990, 33, 2231–2239. 2165165 10.1021/jm00170a030

[6] F. E. Held , K. A. Stingl , S. B. Tsogoeva , Symmetry 2017, 9, 88.

[7] G. Sipos , E. E. Drinkel , R. Dorta , Chem. Soc. Rev. 2015, 44, 3834–3860. 25954773 10.1039/c4cs00524d

[8] E. Wojaczyńska , J. Wojaczyński , Chem. Rev. 2010, 110, 4303–4356. 20415478 10.1021/cr900147h

[9] J. Han , V. A. Soloshonok , K. D. Klika , J. Drabowicz , A. Wzorek , Chem. Soc. Rev. 2018, 47, 1307–1350. 29271432 10.1039/c6cs00703a

[10] T. Toru , C. Bolm , Organosulfur Chemistry in Asymmetric Synthesis, Wiley-VCH, Weinheim 2008.

[11] G. D. Gonzalo , D. E. Torres Pazmiño , G. Ottolina , M. W. Fraaije , G. Carrea , Tetrahedron: Asymmetry 2006, 17, 130–135.

[12] S. Colonna , N. Gaggero , G. Carrea , P. Pasta , V. Alphand , R. Furstoss , Chirality 2001, 13, 40–42. 11135413 10.1002/1520-636X(2001)13:1<40::AID-CHIR8>3.0.CO;2-M

[13] G. Gonzalo , G. Ottolina , F. Zambianchi , M. W. Fraaije , G. Carrea , J. Mol. Catal. B 2006, 39, 91–97.

[14] W. Maczka , K. Wińska , M. Grabarczyk , Catalysts 2018, 8, 624.

[15] F. Garzón-Posse , L. Becerra-Figueroa , J. Hernández-Arias , D. Gamba-Sánchez , Molecules 2018, 23, 1265. 10.3390/molecules23061265 PMC6099930 29799483

[16] H. Y. Kim , V. N. Gladyshev , Biochem. Biophys. Res. Commun. 2004, 320, 1277–1283. 15249228 10.1016/j.bbrc.2004.06.078

[17] M. Reiterer , R. Schmidt-Kastner , S. L. Milton , Free Radical Res. 2019, 53, 1144–1154. 31775527 10.1080/10715762.2019.1662899

[18] C. Achilli , A. Ciana , G. Minetti , BioFactors 2015, 41, 135–152. 25963551 10.1002/biof.1214

[19] M. A. Bennett , Sulfur Metab. 1939, 3, 1794–1797.

[20] M. A. Rahman , H. Nelson , H. Weissbach , N. Brot , J. Biol. Chem. 1992, 267, 15549–15551. 1386361

[21] J. Moskovitz , H. Weissbach , N. Brot , Proc. Natl. Acad. Sci. USA 1996, 93, 2095–2099. 8700890 10.1073/pnas.93.5.2095 PMC39915

[22] Y. Chen , J. Zhuo , D. Zheng , S. Tian , Z. Li , J. Mol. Catal. B 2014, 106, 100–104.

[23] J. Yang , Z. Yuan , Y. Zhou , J. Zhao , M. Yang , X. Cheng , G. Ou , Y. Chen , J. Mol. Catal. B 2016, 133, S588–S592.

[24] C. Achilli , A. Ciana , G. Minetti , Tetrahedron Lett. 2017, 58, 4781–4782.

[25] L. Peng , Y. Wen , Y. Chen , Z. Yuan , Y. Zhou , X. Cheng , Y. Chen , J. Yang , ChemCatChem 2018, 10, 3284–3290.

[26] J. Yang , Y. Wen , L. Peng , Y. Chan , X. Cheng , Y. Chen , Org. Biomol. Chem. 2019, 17, 3381–3388. 30860233 10.1039/c9ob00384c

[27] L. Tarrago , V. N. Gladyshev , Biochemistry 2012, 77, 1097–1107. 23157290 10.1134/S0006297912100021

[28] Y. Wen , L. Peng , Y. Zhou , T. Peng , Y. Chen , X. Cheng , Y. Chen , J. Yang , Catal. Commun. 2020, 136, 1–6.

[29] V. Nosek , J. Míšek , Angew. Chem. 2018, 130, 9997–10000;

[30] J. H. Weiner , R. A. Rothery , D. Sambasivarao , C. A. Trieber , Biochim. Biophys. Acta Bioenerg. 1992, 1102, 1–18. 10.1016/0005-2728(92)90059-b 1324728

[31] H. Ando , M. Kumagai , T. Karashimada , H. Iida , Jpn. J. Microbiol. 1957, 1, 335–338. 13562966 10.1111/j.1348-0421.1957.tb00032.x

[32] P. T. Bilous , J. H. Weiner , J. Bacteriol. 1985, 162, 1151–1155. 3888958 10.1128/jb.162.3.1151-1155.1985 PMC215896

[33] M. Abo , M. Tachibana , A. Okubo , S. Yamazaki , Biosci. Biotechnol. Biochem. 1994, 58, 596–597.

[34] M. Abo , M. Tachibana , A. Okubo , S. Yamazaki , Bioorg. Med. Chem. 1995, 3, 109–112. 7796044 10.1016/0968-0896(95)00004-z

[35] M. Abo , M. Dejima , F. Asano , A. Okubo , S. Yamazaki , Tetrahedron: Asymmetry 2000, 11, 823–828.

[36] S. P. Hanlon , D. L. Graham , P. J. Hogan , R. A. Holt , C. D. Reeve , A. L. Shaw , A. G. McEwan , Microbiology 1998, 144, 2247–2253. 9720047 10.1099/00221287-144-8-2247

[37] H. R. Luckarift , H. Dalton , N. D. Sharma , D. R. Boyd , R. A. Holt , Appl. Microbiol. Biotechnol. 2004, 65, 678–685. 15322772 10.1007/s00253-004-1667-6

[38] N. Makukhin , V. Havelka , E. Poláchová , P. Rampírová , V. Tarallo , K. Strisovsky , J. Míšek , FEBS J. 2019, 286, 4024–4035. 31166082 10.1111/febs.14951

[39] V. Nosek , J. Míšek , Chem. Commun. 2019, 55, 10480–10483. 10.1039/c9cc05470g 31411608

[40] K. I. Chen , V. L. Challinor , L. Kielmann , P. C. Sharpe , J. J. De Voss , U. Kappler , A. G. McEwan , P. V. Bernhardt , J. Biol. Inorg. Chem. 2015, 20, 395–402. 25410832 10.1007/s00775-014-1215-5

[41] M. Tudorache , S. Nica , E. Bartha , I. Lupan , V. I. Parvulescu , Appl. Catal. A 2012, 441–442, 42–46.

[42a] S. Park , R. J. Kazlauskas , Curr. Opin. Biotechnol. 2003, 14, 432–437; 12943854 10.1016/s0958-1669(03)00100-9

[42b] F. van Rantwijk , R. A. Sheldon , Chem. Rev. 2007, 107, 2757–2785; 17564484 10.1021/cr050946x

[42c] N. Kaftzik , P. Wasserscheid , U. Kragl , Org. Process Res. Dev. 2002, 6, 553–557.

[43] Z. Yang , W. Pan , Enzyme Microb. Technol. 2005, 37, 19–28.

[44] N. V. Plechkova , K. R. Seddon , Chem. Soc. Rev. 2008, 37, 123–150. 18197338 10.1039/b006677j

[45] T. Welton , Chem. Rev. 1999, 99, 2071–2083. 11849019 10.1021/cr980032t

[46] A. Zaks , A. M. Klibanov , Proc. Natl. Acad. Sci. USA 1985, 82, 3192–3196. 3858815 10.1073/pnas.82.10.3192 PMC397741

[47] A. Zaks , A. M. Klibanov , J. Biol. Chem. 1988, 263, 3194–3201. 3277967

[48] S. G. Cull , J. D. Holbrey , V. Vargas-Mora , K. R. Seddon , G. J. Lye , Biotechnol. Bioeng. 2000, 69, 227–233. 10861402

[49] S. H. Schöfer , N. Kaftzik , P. Wasserscheid , U. Kragl , Chem. Commun. 2001, 425–426.

[50] R. Madeira Lau , F. Van Rantwijk , K. R. Seddon , R. A. Sheldon , Org. Lett. 2000, 2, 4189–4191. 11150196 10.1021/ol006732d

[51] H. Zhao , S. V. Malhotra , Biotechnol. Lett. 2002, 24, 1257–1259.

[52] N. Kaftzik , P. Wasserscheid , U. Kragl , Org. Process Res. Dev. 2002, 6, 553–557.

[53] K. Okrasa , E. Guibé-Jampel , M. Therisod , Tetrahedron: Asymmetry 2003, 14, 2487–2490.

[54] C. Chiappe , L. Neri , D. Pieraccini , Tetrahedron Lett. 2006, 47, 5089–5093.

[55] R. J. Lichtenecker , W. Schmid , Monatsh. Chem. 2009, 140, 509–512.

[56] P. Gao , A. Li , H. H. Lee , D. I. C. Wang , Z. Li , ACS Catal. 2014, 4, 3763–3771.

[57] F. Gao , L. Wang , Y. Liu , S. Wang , Y. Jiang , M. Hu , S. Li , Q. Zhai , Biochem. Eng. J. 2015, 93, 243–249.

[58] A. P. Abbott , G. Capper , D. L. Davies , H. L. Munro , R. K. Rasheed , V. Tambyrajah , Chem. Commun. 2001, 1, 2010–2011. 10.1039/b106357j 12240264

[59] A. P. Abbott , D. Boothby , G. Capper , D. L. Davies , R. K. Rasheed , J. Am. Chem. Soc. 2004, 126, 9142–9147. 15264850 10.1021/ja048266j

[60] E. L. Smith , A. P. Abbott , K. S. Ryder , Chem. Rev. 2014, 114, 11060–11082. 25300631 10.1021/cr300162p

[61] A. P. Abbott , G. Capper , D. L. Davies , R. K. Rasheed V Tambyrajah , Chem. Commun. 2003, 9, 70–71. 10.1039/b210714g 12610970

[62] Y. H. Choi , J. van Spronsen , Y. Dai , M. Verberne , F. Hollmann , I. W. C. E. Arends , G. J. Witkamp , R. Verpoorte , Plant Physiol. 2011, 156, 1701–1705. 21677097 10.1104/pp.111.178426 PMC3149944

[63] A. Paiva , R. Craveiro , I. Aroso , M. Martins , R. L. Reis , A. R. C. Duarte , ACS Sustainable Chem. Eng. 2014, 2, 1063–1071.

[64] P. Xu , G. W. Zheng , M. H. Zong , N. Li , W. Y. Lou , Bioresour. Bioprocess. 2017, 4. 10.1186/s40643-017-0165-5 PMC5522511 28794956

[65] M. Pätzold , S. Siebenhaller , S. Kara , A. Liese , C. Syldatk , D. Holtmann , Trends Biotechnol. 2019, 37, 943–959. 31000203 10.1016/j.tibtech.2019.03.007

[66] J. T. Gorke , F. Srienc , R. J. Kazlauskas , Chem. Commun. 2008, 1235–1237. 10.1039/b716317g 18309428

[67] D. Lindberg , M. de la Fuente Revenga , M. Widersten , J. Biotechnol. 2010, 147, 169–171. 20438773 10.1016/j.jbiotec.2010.04.011

[68] H. Zhao , G. A. Baker , S. Holmes , J. Mol. Catal. B 2011, 72, 163–167. 10.1016/j.molcatb.2011.05.015 PMC3167219 21909232

[69] H. R. Lobo , B. S. Singh , G. S. Shankarling , Green Chem. Lett. Rev. 2012, 5, 487–533.

[70] Y. Li , Y. Ma , P. Li , X. Zhang , D. Ribitsch , M. Alcalde , F. Hollmann , Y. Wang , ChemPlusChem 2020, 85, 254–257. 31951316 10.1002/cplu.201900751

